# Supplementary material for: Broad Epigenetic Shifts in the Aging Drosophila Retina Contribute to Its Altered Diurnal Rhythmic Transcriptome
Source: Aging Cell. 2026 Feb 12;25(2):e70396. doi: 10.1111/acel.70396 (PMC12895490; doi:10.1111/acel.70396)
Supplement: Supplementary file 1 — Appendix S1: acel70396‐sup‐0001‐AppendixS1.pdf. [file ACEL-25-e70396-s002.pdf]

SUPPLEMENTAL FIGURES

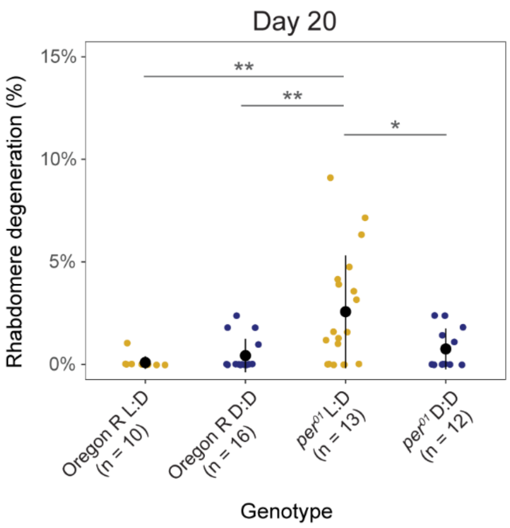

Supplemental Figure 1: Functional *period* is required for protection against light-dependent retinal degeneration.

Optical neutralization of *per* mutant flies relative to the wild-type control, OregonR. Flies were raised in 12-hr:12-hr light:dark (L:D) or dark:dark (D:D) conditions as indicated. * $p < 0.05$, ** $p < 0.01$, *** $p < 0.001$, ANOVA + Tukey's HSD. Number of biological replicates (n) is indicated per condition.

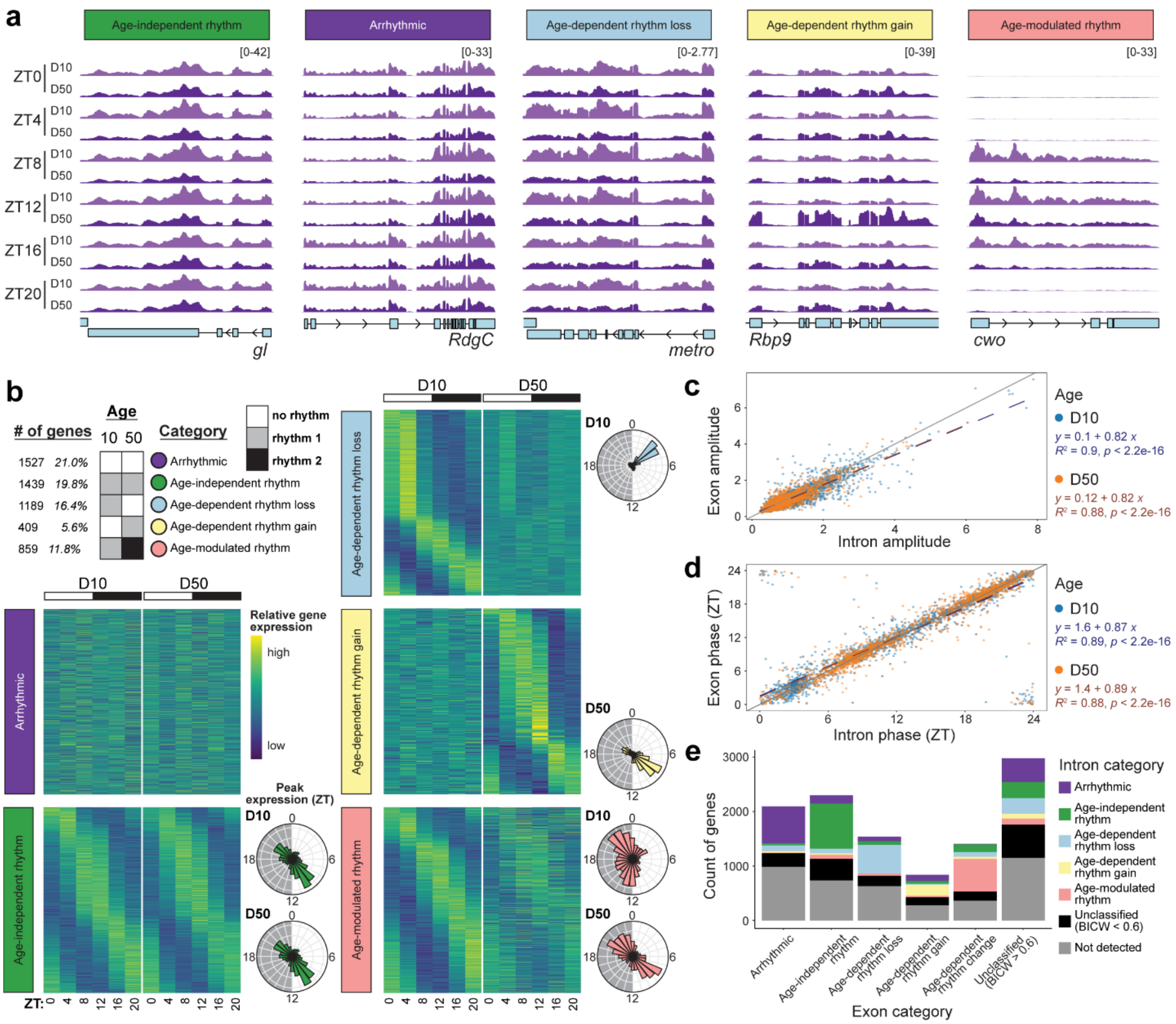

Supplemental Figure 2: Rhythmic patterns of photoreceptor gene expression reflect nascent transcription.

a, Nuclear RNA-seq data includes both intronic and exonic reads, representing nascent transcription. Tracks showing CPM-normalized RNA-seq across all ZTs at both ages for single gene examples from each aging rhythmic gene expression category. Data is mean (n = 3). Exons are indicated by solid boxes and introns by connecting lines for the selected genes, labeled under each track. Arrows indicate direction of transcription. **b,** Rhythmic dryR intron-based categories (BICW ≥ 0.6). Heatmaps show z score of relative expression, and radial histograms show phase of peak gene expression. **c,d,** Scatterplots comparing amplitude (**c**) or phase (**d**) using intron versus exon counts at D10 or D50. Linear regression lines (dashed) are shown for each dataset, with equations, R^2, and p values displayed. The gray solid line represents the expected 1:1 relationship for reference. **e,** Bar plot showing the number of genes in each age-dependent rhythmicity category determined by exonic versus intronic counts. “Not detected” indicates no intronic reads were unambiguously mapped to that gene.

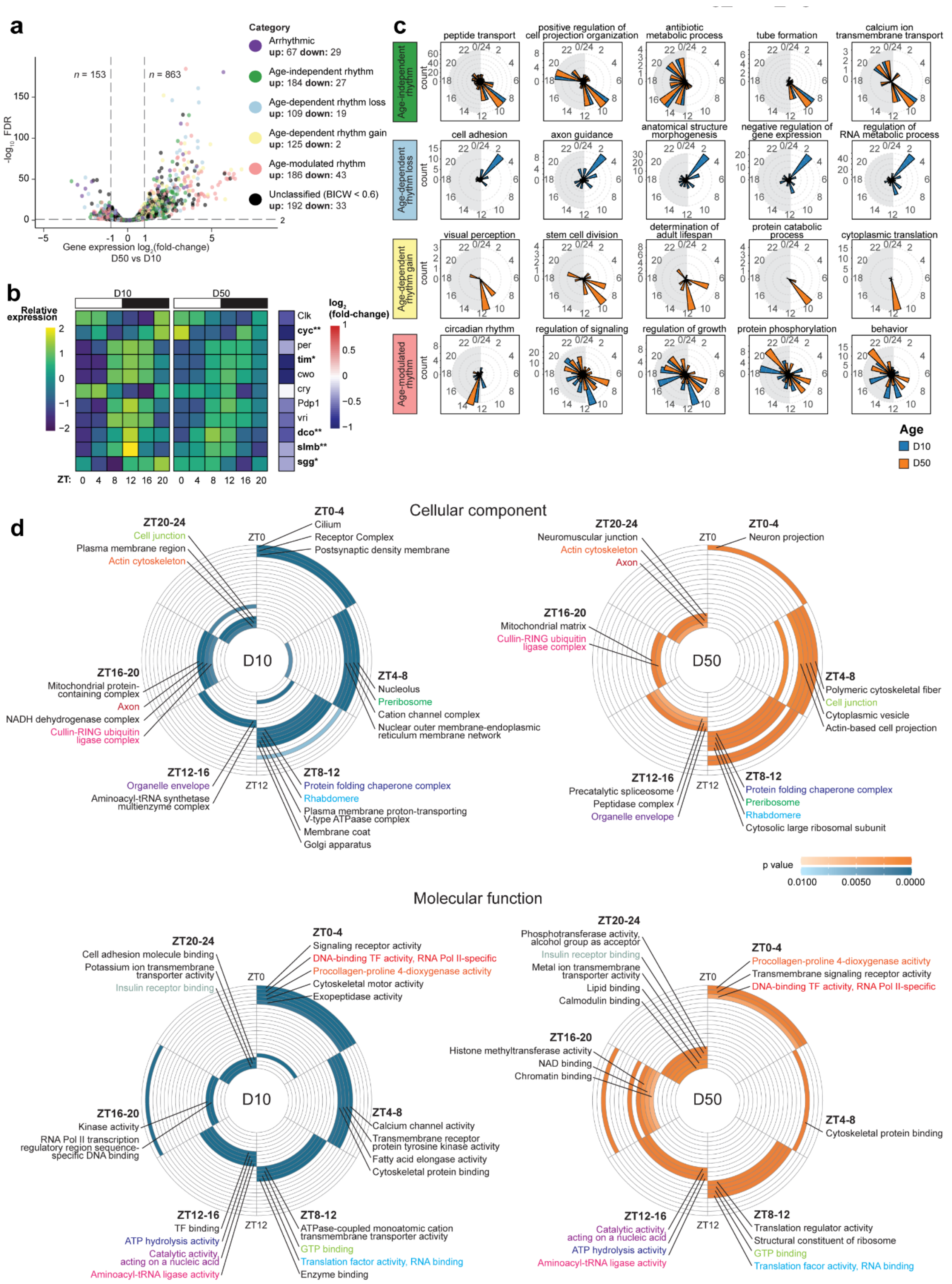
a
Category
Arrhythmic up: 67 down: 29
Age-independent rhythm up: 184 down: 27
Age-dependent rhythm loss up: 109 down: 19
Age-dependent rhythm gain up: 125 down: 2
Age-modulated rhythm up: 186 down: 43
Unclassified (BICW < 0.6) up: 192 down: 33
n = 153
n = 863
-log10 FDR
Gene expression log2(fold-change) D50 vs D10
b
Relative expression
D10
D50
log2 (fold-change)
Clk
cyc**
per
tim*
cwo
cry
Pdp1
vri
dco**
slmb**
sgg*
ZT: 0 4 8 12 16 20 0 4 8 12 16 20
c
Age-independent rhythm
Age-dependent rhythm loss
Age-dependent rhythm gain
Age-modulated rhythm
peptide transport
positive regulation of cell projection organization
antibiotic metabolic process
tube formation
calcium ion transmembrane transport
cell adhesion
axon guidance
anatomical structure morphogenesis
negative regulation of gene expression
regulation of RNA metabolic process
visual perception
stem cell division
determination of adult lifespan
protein catabolic process
cytoplasmic translation
circadian rhythm
regulation of signaling
regulation of growth
protein phosphorylation
behavior
count
Age
D10
D50
d
Cellular component
ZT0-4 Cilium Receptor Complex Postsynaptic density membrane
ZT20-24 Cell junction Plasma membrane region Actin cytoskeleton
ZT16-20 Mitochondrial protein-containing complex Axon NADH dehydrogenase complex Cullin-RING ubiquitin ligase complex
ZT12-16 Organelle envelope Aminoacyl-tRNA synthetase multienzyme complex
ZT8-12 Protein folding chaperone complex Rhabdomere Plasma membrane proton-transporting V-type ATPase complex Membrane coat Golgi apparatus
ZT4-8 Nucleolus Preribosome Cation channel complex Nuclear outer membrane-endoplasmic reticulum membrane network
D10
ZT20-24 Neuromuscular junction Actin cytoskeleton Axon
ZT0-4 Neuron projection
ZT16-20 Mitochondrial matrix Cullin-RING ubiquitin ligase complex
ZT4-8 Polymeric cytoskeletal fiber Cell junction Cytoplasmic vesicle Actin-based cell projection
ZT12-16 Precatalytic spliceosome Peptidase complex Organelle envelope
ZT8-12 Protein folding chaperone complex Preribosome Rhabdomere Cytosolic large ribosomal subunit
D50
p value 0.0100 0.0050 0.0000
Molecular function
ZT20-24 Cell adhesion molecule binding Potassium ion transmembrane transporter activity Insulin receptor binding
ZT0-4 Signaling receptor activity DNA-binding TF activity, RNA Pol II-specific Procollagen-proline 4-dioxygenase activity Cytoskeletal motor activity Exopeptidase activity
ZT16-20 Kinase activity RNA Pol II transcription regulatory region sequence-specific DNA binding
ZT4-8 Calcium channel activity Transmembrane receptor protein tyrosine kinase activity Fatty acid elongase activity Cytoskeletal protein binding
ZT12-16 TF binding ATP hydrolysis activity Catalytic activity, acting on a nucleic acid Aminoacyl-tRNA ligase activity
ZT8-12 ATPase-coupled monoatomic cation transmembrane transporter activity GTP binding Translation factor activity, RNA binding Enzyme binding
ZT20-24 Phosphotransferase activity, alcohol group as acceptor Insulin receptor binding Metal ion transmembrane transporter activity Lipid binding Calmodulin binding
ZT0-4 Procollagen-proline 4-dioxygenase activity Transmembrane signaling receptor activity DNA-binding TF activity, RNA Pol II-specific
ZT16-20 Histone methyltransferase activity NAD binding Chromatin binding
ZT4-8 Cytoskeletal protein binding
ZT12-16 Catalytic activity acting on a nucleic acid ATP hydrolysis activity Aminoacyl-tRNA ligase activity
ZT8-12 Translation regulator activity Structural constituent of ribosome GTP binding Translation facor activity, RNA binding

Supplemental Figure 3: Age-dependent changes in rhythmicity do not necessarily reflect overall changes in gene expression levels.

a, Differential gene expression between D10 and D50 across all ZTs was determined using edgeR, identifying 853 significantly age-upregulated genes and 153 age-downregulated genes (FDR < 0.01, $\log_2$(fold-change) ≥ 1 or ≤ -1). Individual genes are plotted as points on the volcano plot, colored by the age-dependent rhythmicity category. Numbers of overlapping genes for each comparison are shown in the legend. **b,** Heatmaps showing *z* score of relative expression for core clock genes (left) and $\log_2$(fold-change) from D10 to D50 (right). Adjusted *p* values were determined using edgeR. * $p < 0.05$, ** $p < 0.01$. **c,** Selected GO term analysis of genes in each age-dependent rhythmicity category displayed as radial histograms indicating peak gene expression phase at each age. **d,** Selected GO terms of rhythmic genes expressed most highly in four-hour intervals at D10 and D50 for cellular component (top) and molecular function (bottom). GO terms are colored by *p* value within the circles.

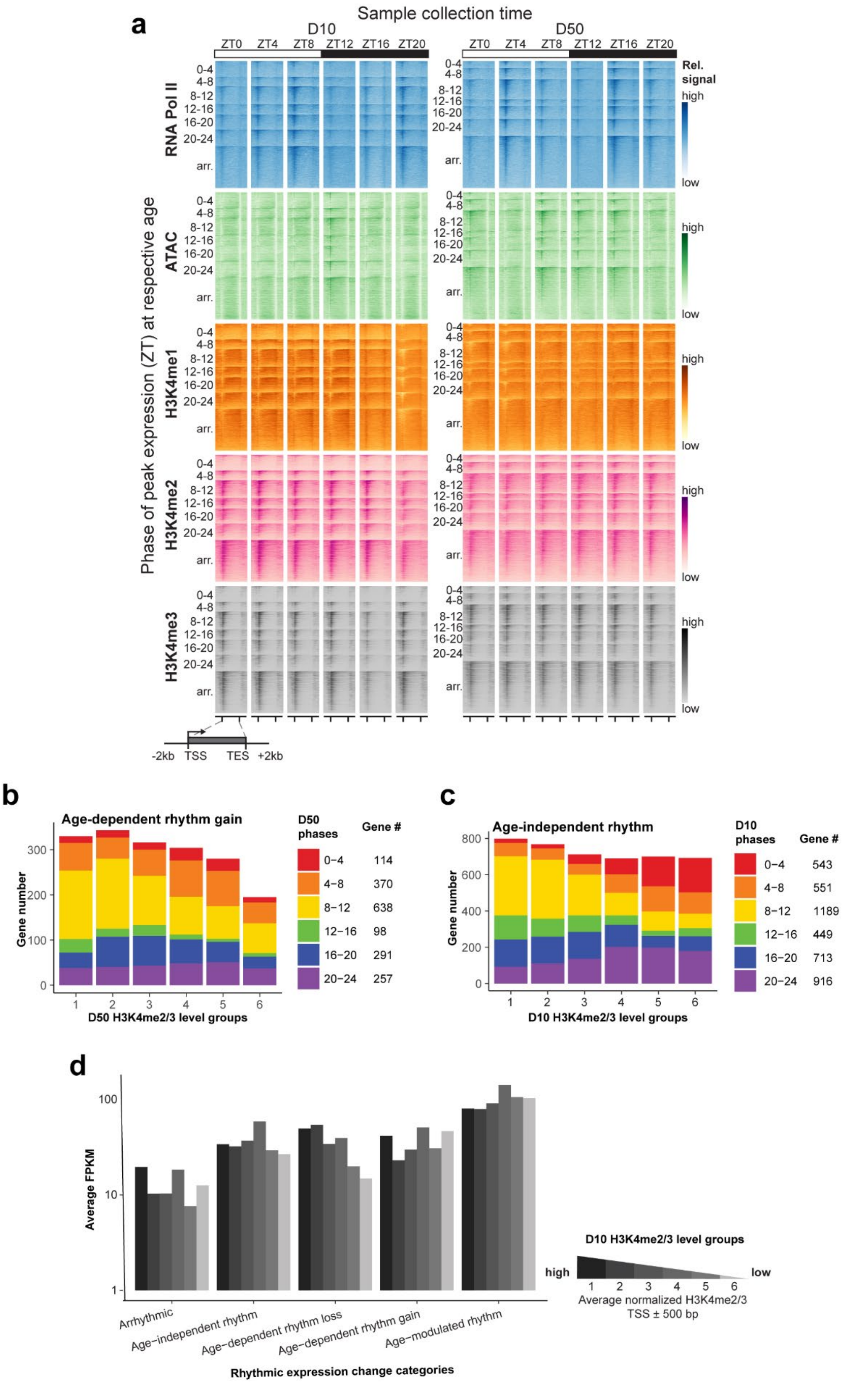

a
Sample collection time
D10
D50
ZT0 ZT4 ZT8 ZT12 ZT16 ZT20
Rel. signal
high
low
RNA Pol II
ATAC
H3K4me1
H3K4me2
H3K4me3
0-4
4-8
8-12
12-16
16-20
20-24
arr.
Phase of peak expression (ZT) at respective age
-2kb TSS TES +2kb
b
Age-dependent rhythm gain
Gene number
D50 H3K4me2/3 level groups
D50 phases
Gene #
0−4 114
4−8 370
8−12 638
12−16 98
16−20 291
20−24 257
c
Age-independent rhythm
Gene number
D10 H3K4me2/3 level groups
D10 phases
Gene #
0−4 543
4−8 551
8−12 1189
12−16 449
16−20 713
20−24 916
d
Average FPKM
Arrhythmic
Age−independent rhythm
Age−dependent rhythm loss
Age−dependent rhythm gain
Age−modulated rhythm
Rhythmic expression change categories
D10 H3K4me2/3 level groups
high
low
Average normalized H3K4me2/3
TSS ± 500 bp

Supplemental Figure 4: H3K4me2/3 levels differ by both phase of peak gene expression, and age-dependent rhythmicity category.

a, Heatmaps representing gene bodies ± 2 kb by relative signal (*z* score) across the same gene (rows) at each ZT and age. Genes are clustered based on the phase of peak expression at D10 or D50 determined by nuclear RNA levels at each age (ZT: 0-4, 4-8, 8-12, 12-16, 16-20, 20-24, arr. = arrhythmic), and sorted within each cluster based on descending Pol II signal. **b,** Bar plot showing the phase distribution of genes that gain rhythmicity based on their relative H3K4me2 and H3K4me3 levels at D50 and expression phase at D10. Genes were separated into six H3K4me2/3 level groups ranging from highest (group 1) to lowest (group 6) based on their average normalized H3K4me2/3 scores at TSS ± 500 bp. **c,** Bar plot showing genes that maintain rhythmicity during aging, based on their phase distribution at D10. **d,** Bar plot showing average FPKM of genes in each rhythmicity category, grouped by average H3K4me2/3 scores at D10 as in **c**.

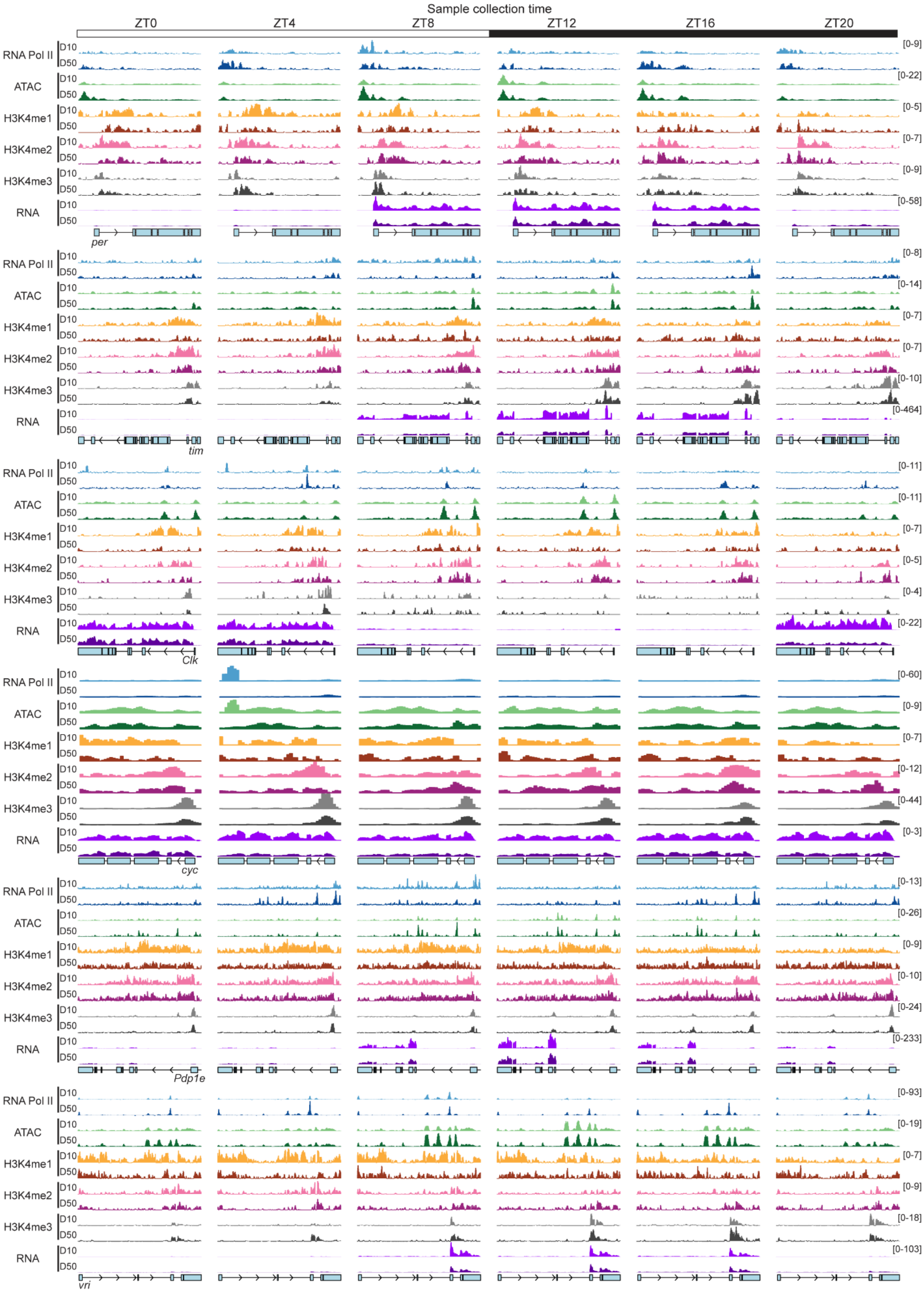

Sample collection time
ZT0
ZT4
ZT8
ZT12
ZT16
ZT20
RNA Pol II
ATAC
H3K4me1
H3K4me2
H3K4me3
RNA
D10
D50
[0-9]
[0-22]
[0-5]
[0-7]
[0-9]
[0-58]
per
[0-8]
[0-14]
[0-7]
[0-7]
[0-10]
[0-464]
tim
[0-11]
[0-11]
[0-7]
[0-5]
[0-4]
[0-22]
Clk
[0-60]
[0-9]
[0-7]
[0-12]
[0-44]
[0-3]
cyc
[0-13]
[0-26]
[0-9]
[0-10]
[0-24]
[0-233]
Pdp1e
[0-93]
[0-19]
[0-7]
[0-9]
[0-18]
[0-103]
vri

Supplemental Figure 5: Certain core clock genes exhibit unique H3K4 methylation oscillation.
ATAC-seq, RNA-seq, and IgG-normalized CUT&RUN tracks at indicated core clock genes across ZTs at D10 versus D50. Data are mean (*n* = 3).

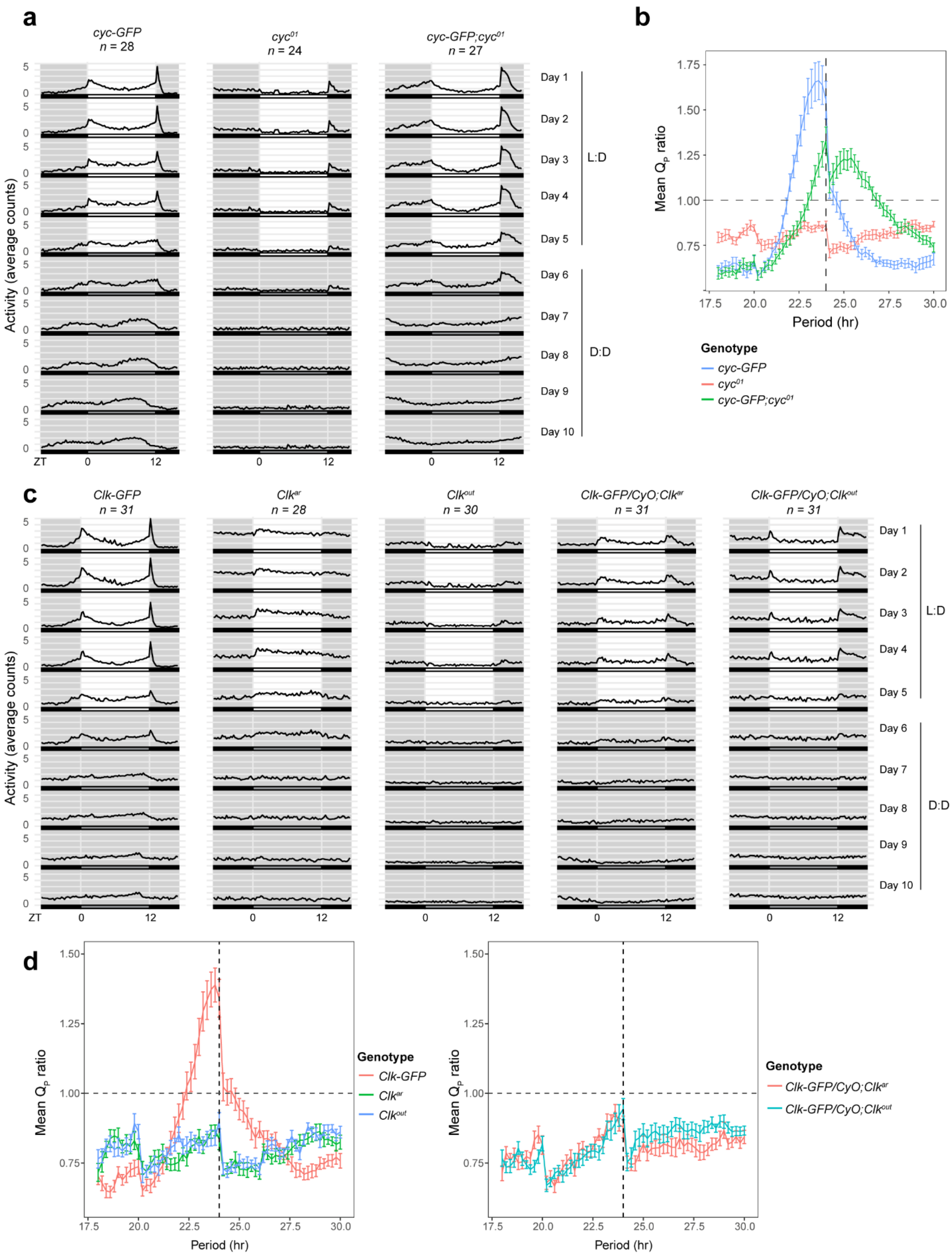
a
cyc-GFP
n = 28
cyc01
n = 24
cyc-GFP;cyc01
n = 27
Activity (average counts)
Day 1
Day 2
Day 3
Day 4
Day 5
Day 6
Day 7
Day 8
Day 9
Day 10
L:D
D:D
ZT
0
12
b
Mean Qp ratio
Period (hr)
Genotype
cyc-GFP
cyc01
cyc-GFP;cyc01
c
Clk-GFP
n = 31
Clkar
n = 28
Clkout
n = 30
Clk-GFP/CyO;Clkar
n = 31
Clk-GFP/CyO;Clkout
n = 31
d
Genotype
Clk-GFP
Clkar
Clkout
Clk-GFP/CyO;Clkar
Clk-GFP/CyO;Clkout

Supplemental Figure 6: The *cyc-GFP*, but not *Clk-GFP*, transgene rescues the circadian locomotor activity rhythms of circadian-deficient flies.

a, Locomotor activity actograms showing mean counts from each genotype (*n* indicated) in 15-minute bins for five days of 12-hr:12-hr L:D followed by five days of complete darkness (D:D). Time of day (ZT) is on the x axis and the background color indicates whether lights are on or off. *cyc-GFP* (BDSC #55838) carries an ectopic GFP-tagged Cyc, cyc^{01} (BDSC #80929) is a *cyc* null line with a premature stop codon in the endogenous *Cyc* gene, and cyc^{01}*;cyc-GFP* is a rescue line homozygous for both the cyc^{01} null allele and *cyc-GFP*. **b,** Chi-square periodogram analysis of locomotor activity rhythms. The ratio of observed to significance threshold periodogram power (Q_P ratio = Q_{act}/Q_{sig}) is plotted as a function of tested period. Each point on the curve represents the mean Q_{act}/Q_{sig} value for a given genotype, with error bars indicating the SEM. The horizontal dashed line denotes the threshold for significant rhythmicity, and the vertical dashed line marks a 24-hr period. **c**, Actograms showing mean counts for each genotype as in **a**. *Clk-GFP* (BDSC #64789) carries an ectopic GFP-tagged Clk; Clk^{out} (BDSC #56754) is a *Clk* null line with a 5398bp deletion; Clk^{ar} (BDSC #24513) is a hypomorphic allele with a single nucleotide substitution; *Clk-GFP/CyO;*Clk^{out} is a hemizygous rescue line for Clk^{out} and *Clk-GFP/CyO;*Clk^{ar} is a hemizygous rescue line for Clk^{ar} . **d,** Periodograms for each indicated genotype.

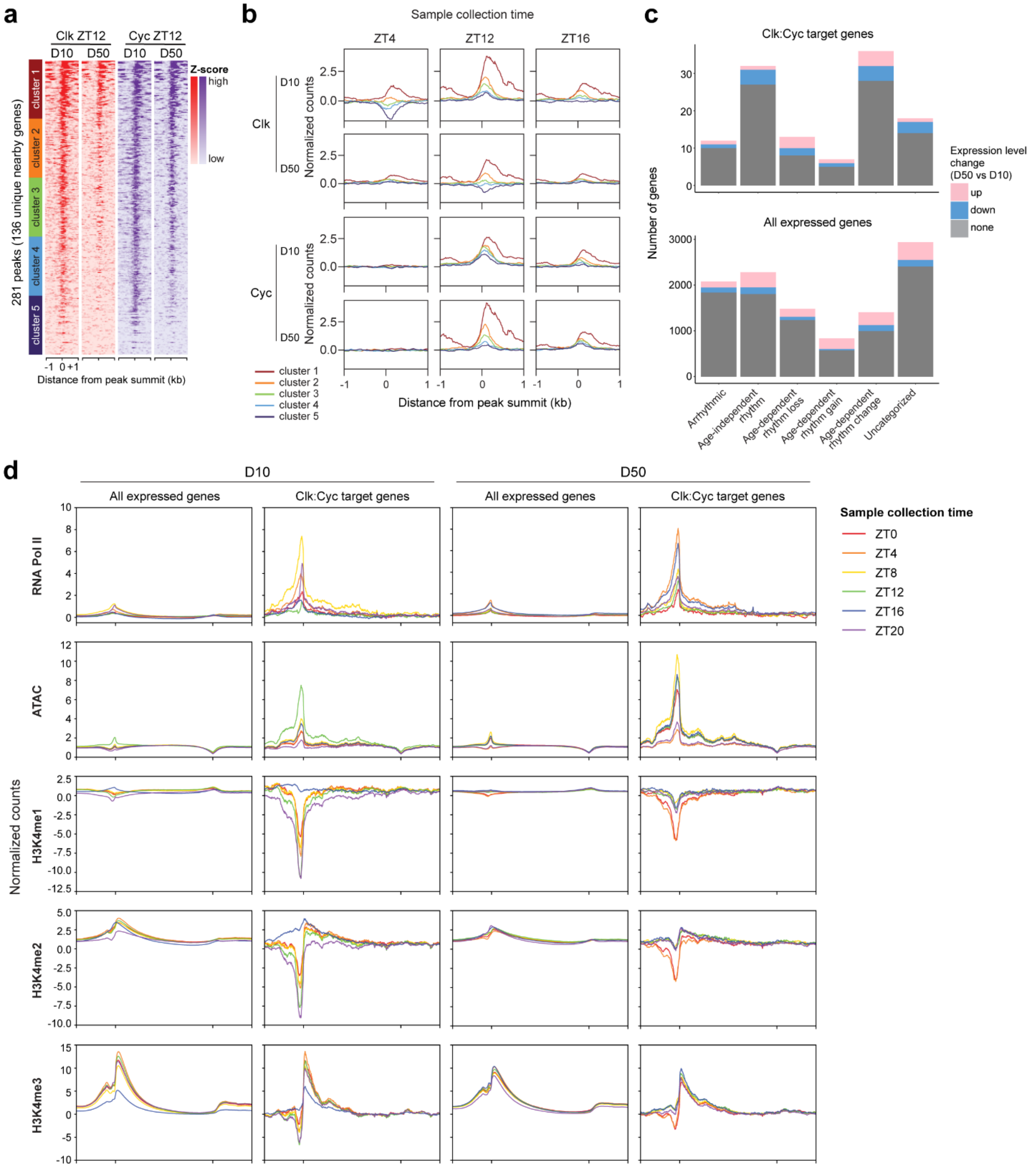
a
Clk ZT12
Cyc ZT12
D10
D50
D10
D50
Z-score
high
low
281 peaks (136 unique nearby genes)
cluster 1
cluster 2
cluster 3
cluster 4
cluster 5
-1 0 +1
Distance from peak summit (kb)
b
Sample collection time
ZT4
ZT12
ZT16
Clk
Cyc
D10
D50
Normalized counts
Distance from peak summit (kb)
c
Clk:Cyc target genes
All expressed genes
Number of genes
Expression level change (D50 vs D10)
up
down
none
Arrhythmic
Age-independent rhythm
Age-dependent rhythm loss
Age-dependent rhythm gain
Age-dependent rhythm change
Uncategorized
d
D10
D50
All expressed genes
Clk:Cyc target genes
RNA Pol II
ATAC
H3K4me1
H3K4me2
H3K4me3
Normalized counts
Sample collection time
ZT0
ZT4
ZT8
ZT12
ZT16
ZT20

Supplemental Figure 7: Clk:Cyc target genes have distinct epigenetic signatures.
a, Heatmaps representing all Clk and Cyc peak centers ± 1 kb colored by relative signal (*z* score) across the same region (rows) ($n = 3$). Heatmaps are sorted by descending Clk signal. Clk:Cyc target genes were separated into six clusters, indicated by colored bars (left), based on high (cluster 1) to low (cluster 6) Clk:Cyc signal. **b,** Gene metaplots representing mean normalized counts for Clk:Cyc peaks in each cluster. Strongest targets (cluster 1) show a distinct pattern of switching from high Clk at D10 to high Cyc at D50. **c,** Bar plot of proportion of Clk:Cyc target genes compared with all expressed genes, colored by age-dependent differential expression (≥ 1.5-fold change, adjusted p value < 0.01, determined using edgeR). **d,** Gene metaplots representing mean normalized counts for CUT&RUN analysis of Pol II, H3K4me1/2/3, and ATAC-seq separated by ZT (collection time for CUT&RUN/ATAC-seq) at D10 versus D50 for all expressed genes compared to 136 Clk:Cyc target genes.

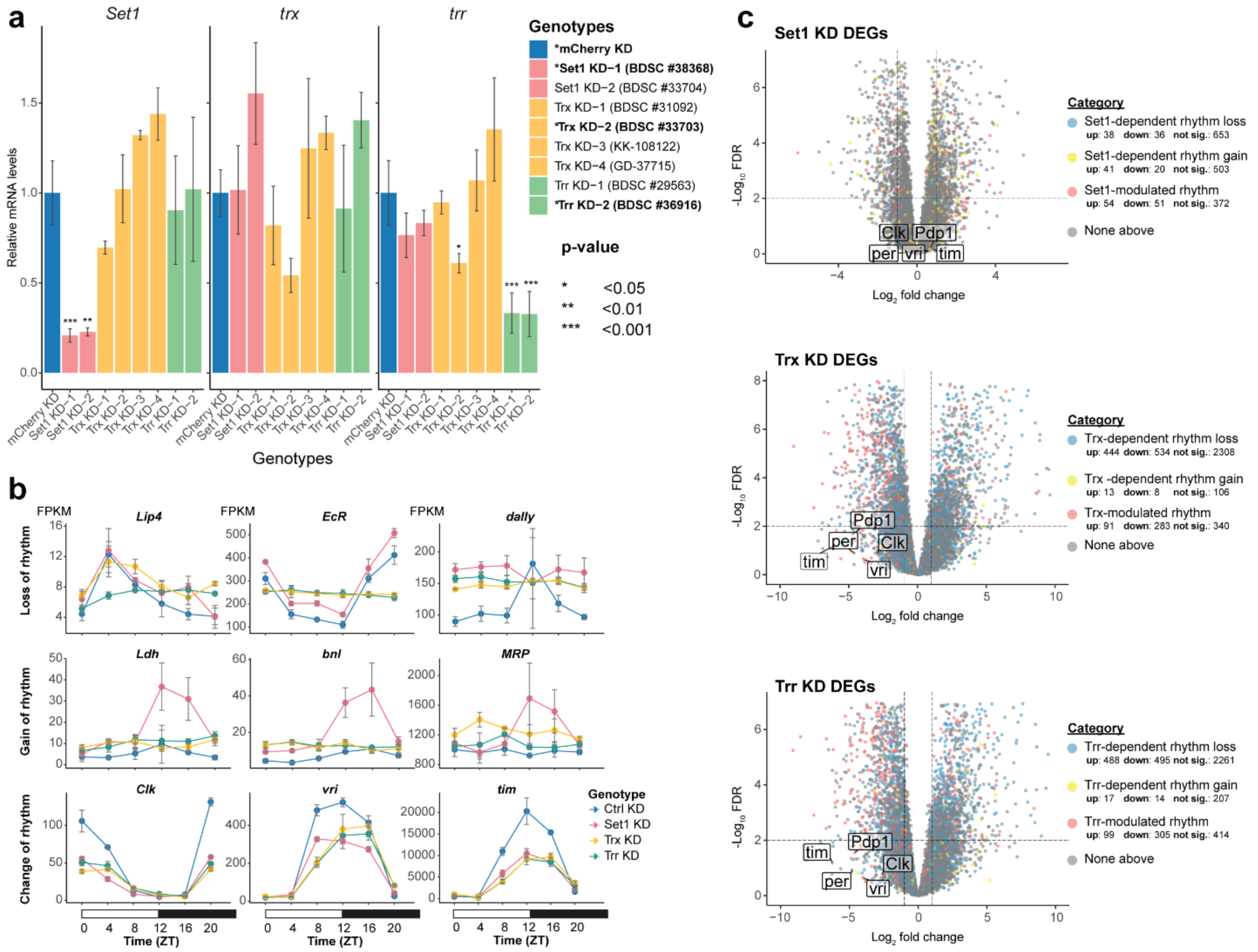

Supplemental Figure 8: HMT-dependent changes in rhythmicity do not necessarily reflect overall changes in gene expression levels.

a, qRT-PCR showing expression of *Set1*, *trr*, *trx* in indicated RNAi lines under *tub-Gal4* control. RNAi expression was restricted using *Gal80*^{ts} by switching third instar larvae from the restrictive (18°C) to permissive (29°C) environment for 72 hours. Conditional knockdown was required because constitutive expression of some RNAi lines was lethal at the larval stage. Fly lines used in this study for further RNA-seq and/or CUT&RUN analysis are indicated in bold and marked with an asterisk. Relative mRNA levels (expression) were calculated as the geometric mean of starting quantity normalized to *Ribosomal protein L32* (*RpL32*) and *eukaryotic translation initiation factor 1A* (*eIF1A*). Bar graphs show relative expression mean ± s.d. (n = 3). ** $p < 0.01$, Dunn's test. **b,** Line plots of gene expression levels for representative genes in the rhythmicity categories. Data are mean FPKM ± s.d. (n = 3). **c,** Differential gene expression between Set1, Trx-, or Trr- KD vs Ctrl KD across all ZTs was determined using edgeR (FDR < 0.01, $\log_2$(fold-change) ≥ 1 or ≤ -1). Individual genes are plotted as points on the volcano plot, colored by the KD rhythmic gene expression category. Numbers of overlapping genes for each comparison are shown in the legend. Select core clock genes are labeled.

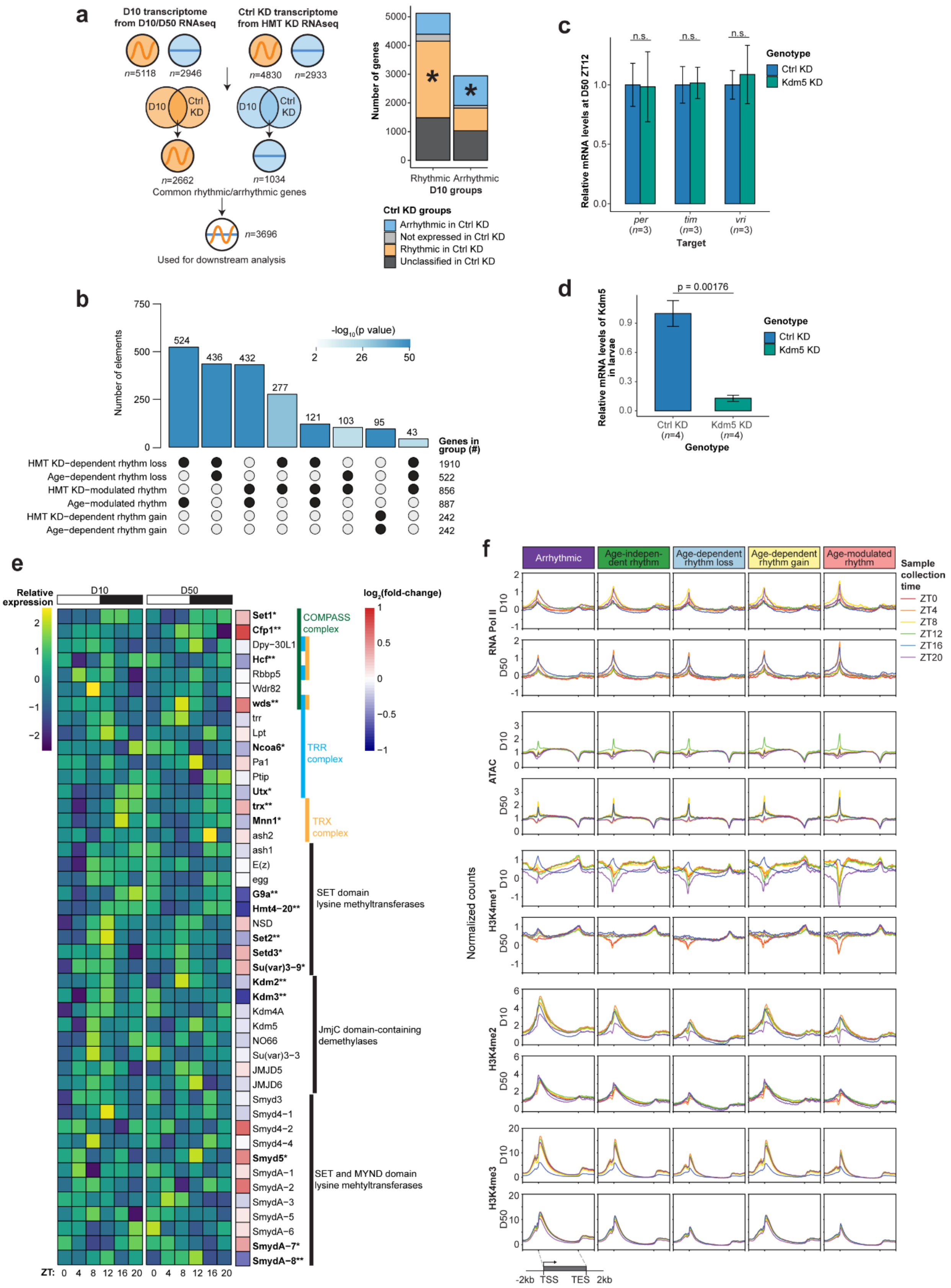
a
D10 transcriptome from D10/D50 RNAseq
Ctrl KD transcriptome from HMT KD RNAseq
n=5118
n=2946
n=4830
n=2933
n=2662
n=1034
Common rhythmic/arrhythmic genes
n=3696
Used for downstream analysis
Number of genes
Rhythmic Arrhythmic
D10 groups
Ctrl KD groups
Arrhythmic in Ctrl KD
Not expressed in Ctrl KD
Rhythmic in Ctrl KD
Unclassified in Ctrl KD
b
Number of elements
-log10(p value)
Genes in group (#)
HMT KD−dependent rhythm loss
Age−dependent rhythm loss
HMT KD−modulated rhythm
Age−modulated rhythm
HMT KD−dependent rhythm gain
Age−dependent rhythm gain
c
Relative mRNA levels at D50 ZT12
Genotype
Ctrl KD
Kdm5 KD
Target
d
Relative mRNA levels of Kdm5 in larvae
p = 0.00176
Genotype
e
Relative expression
log2(fold-change)
COMPASS complex
TRR complex
TRX complex
SET domain lysine methyltransferases
JmjC domain-containing demethylases
SET and MYND domain lysine methyltransferases
f
Arrhythmic
Age-independent rhythm
Age-dependent rhythm loss
Age-dependent rhythm gain
Age-modulated rhythm
Sample collection time
RNA Pol II
ATAC
H3K4me1
H3K4me2
H3K4me3
Normalized counts
-2kb TSS TES 2kb

Supplemental Figure 9: There is no overall trend in gene expression changes of histone methyltransferases and demethylases during aging that explains the observed decreases in histone methylation during aging.

a, Workflow (left) to select genes for comparison between aging and HMT (Set1, trr, trx) KD RNA-seq. Genes selected for downstream analysis were required to have rhythmicity or to be arrhythmic in both controls. Bar plots (right) show the number of genes falling into different rhythmicity categories between the two RNA-seq experiments. Asterisks mark the groups of genes that were used in the downstream analysis. **b,** UpSet plot describing the number of overlapping genes between the aging and HMT knockdown rhythmicity groups. Groups included in comparison are marked with black dots at the bottom, and bar chart on the top shows the number of overlapping genes, colored by statistical significance of intersections. HMT KD encompasses the combination of all three methyltransferase knockdown datasets. **c,** qRT-PCR from adult *Drosophila* heads showing expression of *per*, *tim,* and *vri* in indicated RNAi lines under *Rh1-Gal4* control. Relative mRNA levels (expression) were calculated as the geometric mean of starting quantity normalized to *Ribosomal protein L32* (*RpL32*) and *eukaryotic translation initiation factor 1A* (*eIF1A*). Bar graphs show relative expression mean ± s.d. Sample number (*n*) is indicated. *p* values were determined using Student's t-test. **d,** qRT-PCR from *Drosophila* wandering third-instar larvae showing expression of *Kdm5* in indicated RNAi lines under *tub-Gal4* control. Bar graphs show relative expression mean ± s.d and were normalized as in **c**. Sample number (*n*) is indicated. *p* values were determined using Student's t-test. **e,** Heatmaps showing *z* score of relative gene expression (left) and $\log_2$(fold-change) from D10 to D50 (right) for selected histone methyltransferases, complex subunits, and histone demethylases. Complexes were based on FlyBase annotations. Adjusted *p* values were determined using DESeq2. * $p < 0.05$, ** $p < 0.01$. **f,** Gene metaplots representing mean normalized counts at D10 or D50 for Pol II, H3K4me1/2/3, and ATAC-seq at each of the aging rhythmic gene expression categories (columns) ($n = 3$). Lines are colored by phase of peak gene expression at the respective age.
